# Reconstruction of Mandibular Defects Using Bone Morphogenic Protein: Can Growth Factors Replace the Need for Autologous Bone Grafts? A Systematic Review of the Literature

**DOI:** 10.1155/2011/165824

**Published:** 2011-11-24

**Authors:** Alan S. Herford, Enrico Stoffella, Rahul Tandon

**Affiliations:** ^1^Department of Oral and Maxillofacial Surgery, Loma Linda University, Loma Linda, CA 92354, USA; ^2^Department of Dental Implants, University of Milan, Fondazione IRCCS Cà Granda, Ospedale Maggiore Policlinico, 20122 Milan, Italy

## Abstract

Autogenous bone is still considered the “gold standard” of regenerative and reconstructive procedures involving mandibular defects. However, harvesting of this material can lead to many complications like increasing morbidity, expanding of the surgical time, and incomplete healing of the donor site. In the last few years many authors looked for the development of effective reconstruction procedures using osteoinductive factors without the need for conventional bone grafting. The first-in-human study involving the use of Bone Morphongenic Proteins (rhBMP) for mandibular reconstruction was performed in 2001 by Moghadam. Only few articles have been reported in the literature since then. The purpose of this study was to search and analyze the literature involving the use of rhBMP for reconstruction of mandibular defects. In all the studies reported, authors agree that the use of grown factors may represent the future of regenerative procedures with more research necessary for confirmation.

## 1. Introduction

Tissue engineering holds great promise for revolutionizing many grafting procedures. Continuity defects of the mandible frequently result from tumor removal or significant trauma, and reconstruction of these defects can be challenging. For defects with extensive hard and soft tissue loss, microvascular free tissue transfer often provides an excellent reconstructive option. However, significant site morbidity as well as non ideal bone stock for dental implant rehabilitation may occur [1].

The development of bone morphogenic proteins (BMPs) has offered an alternative to traditional bone grafting, which has been the gold standard for oral and maxillofacial reconstruction [2]. Clinical application of BMPs has evolved to include defects of the facial skeleton including those involving the mandible and maxilla [3]. There have been many reports of the use of BMPs regarding orthopaedic as well as alveolar augmentation. There have been few studies addressing the use of BMP in reconstructing critical-size defects of the mandible.

The purpose of this study is to evaluate the different study present in the literature concerning the use of growth factors for the reconstruction of mandibular defects, comparing the method and the finals results. A well-identified guideline is in fact not still available and, because of that, significative differences could be find analysing the literature on that topic.

## 2. Background

Failure to adequately restore mandibular continuity defects often result in poor function for the patient postsurgically. Various bone grafting and bone manipulation techniques are available for restoring large mandibular bony defects. An ideal osseous grafting treatment should involve use of a bone inductive material that would be reliable, biocompatible, long-lasting, and capable of restoring mandibular continuity with minimal morbidity. Particulate marrow and cancellous autogenous bone meet these requirements, but must be harvested from a donor site that often results in insufficient bone graft material, at an added cost, and patient harvest-graft-site morbidity [4–6]. The development of effective reconstruction procedures using osteoinductive factors, such as growth factors (GFs), without the need for conventional bone grafting has allowed the possibility to decrease surgical morbidity [7–9]. 

Three vital components necessary for the engineering of bone are bone-forming cells, osteoinductive growth factors, and an osteoconductive scaffold. The osteoconductive scaffold provides immediate mechanical support, mimics the bony extracellular matrix, and guides the formation of bone in the desired shape and place. The main role of the growth hormone is to recruit mesenchymal stem cells to the area and influence them to differentiate into an osteogenic cell lineage [10]. Autogenous bone is considered the gold standard for the reconstruction of the mandibular bone because it owns all these properties. Unfortunately, the risk of morbidity is a concern [11, 12] and has led to the great appeal of protein guided bone regeneration.

Many growth factors are involved in osteogenesis [2, 13]. Bone morphogenetic proteins (BMP-2 and BMP-7), transforming growth factor beta (TGF-*β*), insulin-like growth factors I and II (IGF I and II), platelet-derived growth factor (PDGF), fibroblast growth factors (FGFs), and vascular endothelial growth factor (VEGF) have been proposed for use in bone tissue engineering. Bone morphogenetic proteins are a group of proteins known to play a role in osteogenesis and chondrogenesis. BMP-2 and BMP-7 are known for their osteoinductive qualities [10, 14]. Recombinant human BMP in combination with a collagen sponge carrier made out of type 1 bovine collagen has been approved by the Food and Drug Administration and is used for specific clinical situations, that is, interbody spinal fusion, open tibial fractures, sinus augmentation, and localized alveolar ridge augmentation after dental extraction.

## 3. Materials and Methods

### 3.1. Literature Review

An electronic search was conducted on MEDLINE, EMBASE, and CENTRAL database through PubMed, Embase, and Cochrane. The search was based on two aspects: reconstruction of mandibular defects regardless of aetiology and the use of rhBMP, both to be found in title and/or abstract. For the search of mandibular reconstruction, synonyms were taken into account. The search results were checked for doubles, then the unique search results were checked against our exclusion criteria. Due to the small amount of results, the authors were obligated to take in consideration also studies in which the number of the patient was minimum, including case reports. There are no comparative studies, in which the use of rhBMP is compared with autogenous bone for mandibular continuity defects. The only exclusion criteria used were as follows: study performed in nonhuman models and/or language of the full article other than English. The search yielded 8 results (Table 1).

### 3.2. Full Text Analysis

The articles were evaluated with regard to type of study as well as number of patients treated. The type of BMP used, the size of the defect, the type of carrier, and any graft extender used were determined from the reports. The method of measuring the regenerated bone as well as aetiology of the defect was analyzed. Length of followup, whether dental implants were used to rehabilitate the patient, and complications (no bone formation) were also recorded.

Four of the eight articles considered were published by the same author [1, 3, 4, 15]. In between the other four articles, the first one in terms of date of publishing is by Moghadam et al. [16], followed by Clokie and Sándor [17], Carter et al. [18], and Glied and Kraut [19]. Of the 8 articles taken in consideration 7 used rhBMP 2 for the reconstruction of the mandible with only Clokie and Sándor using rhBMP 7. One other article, proposed by P. Warnke et al. [20] and P. H. Warnke et al. [21] in 2004, describes the mandibular reconstruction using 7 mg of rhBMP 7 and a prefabrication technique. This article has not been considered in our paper because the healing process was not obtained in situ but into the latissimus dorsi muscle and the new reconstructed mandible was then transplanted as a free bone-muscle flap to repair the mandibular defect. This case represents the only case in the literature in which the transplanted technique is used for mandibular reconstruction and for that reason is not comparable with the other studies considered in our paper.

## 4. Results

BMPs were used in combination of an absorbable collagen carrier (ACS) in seven of the eight cases. The only case performed in a different way was done by Clokie and Sándor. They used rhBMP 7 in addition to demineralised bone matrix (DBM). In all the other cases the carrier used was an absorbable collagen sponge (ACS), except for the first case reported in the literature, in which Moghadam et al. used a poloxamer-based gel to create the bioimplant. Carter et al. and Clokie and Sándor used demineralised bone matrix, as well as Herford [1], in addition to rhBMP 2 and ACS. The dose used varied considerably in between the first case, reported by Moghadam and the following ones. Moghadam's dose used was in fact 200 mg [16], while the quantity of BMP reported in the other studies varies in between 4.2 mg [15] and 24 mg [19], so considerably inferior to the first one reported in the literature. The authors' opinion is that the first dose used was excessive but justified by the absence of previous studies. The high cost of rhBMP obligated a significant reduction of the bioimplant's amount used in the most recent studies but it did not seem to influence the reconstruction results.

The most common etiology for the defect was benign neoplasms, with diagnosis of ameloblastoma, juvenile ossifying fibroma, giant cell tumor, or traumatic. In his study Carter described one case of mandible reconstruction following enucleation of an odontogenic keratocyst. The fact that all the cases reported in the literature involved benign neoplasm and not malignant lesions confirms the contraindication in using rhBMP in these kind of pathologies, as suggested by FDA. The size of the reconstructed defect, when reported, varied from 3 to 9 cm. The measurement system to evaluate the size of the defect and the following bone regeneration was in all the cases computed tomography (CT) scan prior to, immediately after surgery and at the final followup. In his study Moghadam also used an incisional biopsy to evaluate the amount of bone regeneration and remodelling. The follow-up period in these studies varied from 2 to 22 months, as reported in Table 2.

To evaluate the final result, all the authors based their analysis on the quantity of regenerated bone in the grafted area. The possibility to perform a prosthetic rehabilitation was considered the key for success in most of the studies. Implant rehabilitation was shown in two of the studies considered. Clokie and Sándor described it in four of his ten patients and Herford, in his work of 2009, in one of two patients. The other studies did not describe any implant rehabilitation but in six of the eight articles CT scans showed adequate height and width to support dental implants. Glied and Kraut described a complete failure of the bioimplant in all of his three patients and the necessity of a following reconstruction using autogenous bone grafts. In two of their five patients furthermore Carter et al. had to resort to a second surgery with autogenous bone graft due to the failure of the rhBMP 2. A total of 37 patients were treated in the reviewed studies: 32 were successful (86.5%), whereas 13.5% failed to form adequate bone with rhBMP-2.

## 5. Case Example

**Figure 1:**
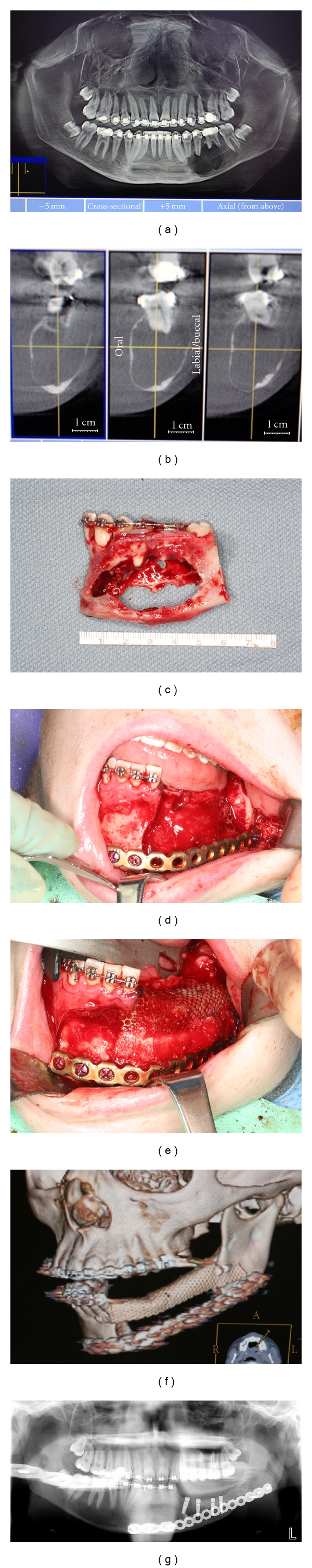
(a) Panoramic radiograph exam shows the presence of a radiolucent lesion in the left mandibular body. The diagnosis was ameloblastoma. (b) CT scan images of the lesion. (c) Tumor specimen, approximately 6 cm. (d) Reconstruction plate in place to reconstruct the mandible. (e) Bioimplant (rhBMP-2/ACS and demineralised bone allograft) inserted and covered by titanium mesh. (f) Postoperatory CT scan. (g) Panoramic radiograph showing implant rehabilitation after 8 months.

A 20-year-old patient presented for evaluation of his mandibular tumor (Figure 1). He had a biopsy performed which revealed an aggressive benign lesion identified an ameloblastoma involving the left mandible. The tumor was resected, and a reconstruction plate was placed. A combination of rhBMP-2 (Infuse Bone Graft—Medironic, TN) and demineralised bone matrix (DBX—Synthes, Paoli, PA) was used to reconstruct the defect. A titanium mesh was placed on the superior aspect of the defect to aid in preventing soft tissue collapse during bone formation (“space maintenance”). The patient was rehabilitated with 4 dental implants after 8 months of healing.

## 6. Discussion

The first reported mandibular reconstruction using rhBMP in a human was reported by Moghadam et al. in 2001 [16]. Over the ten years since the first publication, only a few articles have been reported. The lack of studies does not allow a systematic review with a high level of evidence. The present study in fact, because of the small number of articles available, includes case reports and case reviews. The higher level of evidence in the studies reviewed is obtained by Herford and Boyne [4], who described the treatment of 14 patients with a followup of six months. Clokie and Sándor [17] described 10 cases with a followup of 12 months, Carter et al. [18] 5 cases and 22 months of followup, and Glied and Kraut [19] showed the treatment of 3 patients in 12 months. In his other 3 works Herford [1, 3, 15] described two patients in one article and one in the other two with an average followup of 4 months. Moghadam et al. [16] described one case in his work, with a follow up of 9 months. The total of patients present in the literature, treated with rhBMP for mandibular reconstruction since 2001, is 37. This is a small number if compared with the data present concerning alveolar cleft reconstruction using rhBMP, for which more than 50 studies are reported in the literature [10]. 

rhBMP-2 was used in all case series with the exception of Clokie and Sándor, who used rhBMP 7. In 6 over 7 cases the carrier is an absorbable collagen sponge (ACS). In his study Moghadam used a poloxamer-based gel in addition to rhBMP2, to create the bioimplant. Only three studies [17, 18] utilized demineralised bone matrix, and in one study autogenous bone as a graft extender was used. The addition of a graft extender is recommended in order to promote quicker calcification and better osteoconductivity at the graft site. Another important advantage of adding a graft material is the superior space maintenance associated with it. The ACS is helpful as a carrier and gradually releases the BMP into the defect over the first weeks during osteogenesis. Unfortunately it is easily deformed and it is inadequate in maintaining the space necessary to obtain an optimal bone regeneration.

In spite of these differences, the surgical technique described for the mandibular reconstruction is similar in all the studies considered. Some differences can be found according to the nature of the lesion. 

The analysis of the two studies in which the failures were reported reveals possibilities as to why they failed. In the study by Carter et al., two patients out of five failed to form adequate bone. In the first patient, there was chronic infection with bacteria cultured from the bone at the time of rhBMP-2 placement. Also the reconstruction plate fractured at some point postoperatively and led to mobility of the segiments. The second patient also had osteomyelits with active infection requiring multiple incision and drainage procedures. Both patients exhibited poor compliance and active infection. One of the contraindications for the use of rhBMP-2 is the presence of active infection. In the study by Glied and Kraut, a cadaveric rib was utilized to provide space maintenance for the rhBMP-2. It is possible that this technique provided inadequate framework. It is also possible that the cadaveric rib was insufficiently secured into place thereby producing mobility in the area of the defect. Another patient in their series was a fracture patient with history of osteomyelitis which may have contributed to the minimal bone formation.

## 7. Conclusions

There is general agreement among these authors that osteoinductive materials may be useful in decreasing surgical morbidity and intraoperative time. Even if autogenous bone is still considered the gold standard for reconstruction of mandibular defects, the possibility to obtain the same goal while avoiding autogenous bone harvest is an important aspect to take in consideration.

Based on the small number of studies present in the literature as well as the 13.5% failure rate reported, it is not possible to conclude that growth factors can replace the need for autologous bone grafts at this time. The application of rhBMP with an absorbable sponge collagen appears to be a very promising technique. However, more comparative studies and randomized controlled clinical trials will help to determine the true efficacy of this technique as well as optimal carrier and scaffold.

## Figures and Tables

**Table 1:** Selected articles present in the literature.

Authors	Title	Journals	Years
Moghadam et al. [16]	Successful mandibular reconstruction using a BMP bioimplant	The Journal of Craniofacial Surgery	2001
Herford et al. [3]	Clinical applications of rhBMP-2 in maxillofacial surgery	Journal of the California Dental Association	2007
Clokie and Sándor [17]	Reconstruction of 10 major mandibular defects using bioimplants containing BMP-7	Journal of the Canadian Dental Association	2008
Herford and Boyne [4]	Reconstruction of mandibular continuity defects with bone morphogenetic Protein-2 (rhBMP-2)	Journal and Maxillofacial Surgery	2008
Carter et al. [18]	Off-label use of recombinant human bone morphogenetic protein-2 (rhBMP-2) for reconstruction of mandibular bone defects in humans	Journal and Maxillofacial Surgery	2008
Herford [1]	rhBMP-2 as an option for reconstructing mandibular continuity defects	Journal and Maxillofacial Surgery	2009
Glied and Kraut [19]	Off-label use of rhBMP-2 for reconstruction of critical-sized mandibular defects	New York State Dental Journal	2010
Herford and Cicciù [15]	Recombinant human bone morphogenetic protein type 2 jaw reconstruction in patients affected by giant cell tumor	The Journal of Craniofacial Surgery	2010

**Table 2:** Analysis of the articles.

	Moghadam et al. [16]	Herford et al. [3]	Clokie and Sándor [17]	Herford and Boyne [4]	Carter et al. [18]	Herford [1]	Glied and Kraut [19]	Herford and Cicciù [15]
Type of study	Case report	Case report	Case report	Case review	Case report	Case report	Case report	Case report
No. of patients	1	1	10	14	5	2	3	1
Type of BMP used	Human BMP	rh BMP 2	rh BMP 7	rh BMP 2	rh BMP 2	rh BMP 2	rh BMP 2	rh BMP 2
Size of defect	6 cm	Not declared	3–9 cm	4–8 cm	5 cm	Not declared	4.5–8 cm	Not declared
BMP dose	200 mg	8 mg	n.d.	6 mg	8.4 mg/12 mg	12 mg	24 mg	4.2 mg
Carrier	Poloxamer-based gel	ACS	None	ACS	ACS	ACS	ACS	ACS
Graft extender	None	None	DBM	None	DBM	DBM	None	ICBG
Method of measuring	CT scan + biopsy	CT scan	CT scan	CT scan	CT scan	CT scan	CT scan	CT scan
Followup	9 months	2 months	9–12 months	6 months	8–22 months	8-9 months	6–12 months	4 months
Etiology	Ameloblastoma	Juvenile ossifing fibroma	Ameloblastoma/ Osteomyelitis	Neoplastic disease/ Osteomyelitis	Trauma/ Cyst	Ameloblastoma	Ameloblastoma	Giant cells tumor
Implant rehabilitation	None	None	4 of 10 patients	None	None	1 of 2 patient	None	None
Complications	None	None	None	None	2 patients failed	None	Complete failure	None
